# Total solids content: a key parameter of metabolic pathways in dry anaerobic digestion

**DOI:** 10.1186/1754-6834-6-164

**Published:** 2013-11-22

**Authors:** Jean-Charles Motte, Eric Trably, Renaud Escudié, Jérôme Hamelin, Jean-Philippe Steyer, Nicolas Bernet, Jean-Philippe Delgenes, Claire Dumas

**Affiliations:** 1 INRA, UR0050, Laboratoire de Biotechnologie de l′Environnement, Avenue des Etangs, Narbonne F-11100, France

**Keywords:** Biohydrogen, Dark fermentation, Fermentative metabolites, Moisture content, Lignocellulosic residues

## Abstract

**Background:**

In solid-state anaerobic digestion (AD) bioprocesses, hydrolytic and acidogenic microbial metabolisms have not yet been clarified. Since these stages are particularly important for the establishment of the biological reaction, better knowledge could optimize the process performances by process parameters adjustment.

**Results:**

This study demonstrated the effect of total solids (TS) content on microbial fermentation of wheat straw with six different TS contents ranging from wet to dry conditions (10 to 33% TS). Three groups of metabolic behaviors were distinguished based on wheat straw conversion rates with 2,200, 1,600, and 1,400 mmol.kg_VS_^-1^ of fermentative products under wet (10 and 14% TS), dry (19 to 28% TS), and highly dry (28 to 33% TS) conditions, respectively. Furthermore, both wet and dry fermentations showed acetic and butyric acid metabolisms, whereas a mainly butyric acid metabolism occurred in highly dry fermentation.

**Conclusion:**

Substrate conversion was reduced with no changes of the metabolic pathways until a clear limit at 28% TS content, which corresponded to the threshold value of free water content of wheat straw. This study suggested that metabolic pathways present a limit of TS content for high-solid AD.

## Background

At present, anaerobic digestion (AD) of agricultural waste such as manure and lignocellulosic residues represents an economical, environmental, and societal opportunity to produce massively renewable energy (methane and hydrogen) and agricultural amendment through a digestate of high agronomic quality [1,2]. The AD process is supported by complex microbial ecosystems capable of converting the organic matter into methane and carbon dioxide by the following four steps: hydrolysis, acidogenesis, acetogenesis, and methanogenesis. Nowadays, AD bioprocess systems are mature technologies, with constant development since the 1990s [3]. Process design is mostly dependent on the waste characteristics which constrain the temperature (mesophilic or thermophilic), the configuration (one- or two-stage reactors), or the feeding modes (mono-substrate or codigestion) [3,4]. Water content is known to be one of the most important parameters that can affect the whole process of AD [1,5,6]. Therefore, total solids (TS) content of the medium is usually used to define two types of processes: wet digestion for TS <15% and dry digestion or high-solid TS >15 to 20% [1,3]. Operating AD under dry conditions offers the advantage of reducing reactor size, liquid/solid separation systems, and lowering energy consumption for bioprocess heating [7,8].

Intrinsic advantages of dry AD bioprocesses have led to an early industrial development of digesters operated between 20 and 30% TS and up to 40% TS for some technologies [7]. However, this technical development was mainly based on empirical knowledge. Among the few studies dealing with the effect of TS content, Brown *et al.*[9] showed significant differences in anaerobic kinetics between wet and dry digestion, but no impact on methane yields. The effect of TS content on AD was also reported by Abbassi-Guendouz *et al.*[8] with cardboard and by Staley *et al*. [10] with residential area waste. Both studies reported a similar strong inhibition of AD performances for TS higher than 30% with a failure in methane production through the accumulation of volatile fatty acids (VFAs). The solid-state conditions favored the emergence of specific microorganisms [11]. Abbassi-Guendouz *et al*. [12] observed the emergence of specific fermenting species of *Clostridium* in inhibited systems at high TS content. So far, the effect of TS content on dry microbial fermentation has rarely been evaluated. Only two studies compared the effects of a gradient of TS content (from 21 to 35% TS [13] and from 10 to 35% TS [14]) on biohydrogen production. Both concluded to a decrease of hydrogen production with an increase of TS content and the importance of alkalinity to buffer the media. However, these studies did not evaluate the impact of TS content on the metabolic pathways involved in dry conditions. Therefore, there is an important lack of knowledge on microbial fermentation metabolism related to the water content reduction.

The first two steps of AD, hydrolysis and acidogenesis, are commonly considered as the limiting steps of solid waste degradation [4,10,15]. During these steps, several fermentative microbial pathways can be involved to degrade the organic matter [13]. Microbial fermentation consists in waste degradation to simple molecules that will be further used as substrate by methanogenic consortia [16]. As shown in Table 1, three categories of fermentative end-products are generated when methanogenesis is blocked or inhibited: 1) a gaseous phase composed of hydrogen and carbon dioxide; 2) by-products associated with hydrogen-producing pathways, that is acetic and butyric acid; and 3) other metabolites of non-hydrogen-generating pathways such as propionic, valeric, and caproic acids, ethanol, or lactic acid [16,17]. Since acetic and butyric acids are easier to degrade than other VFAs or organic acids [16,18], their production and distribution after acidogenesis can be very informative on the ability of the system to easily start-up. A butyric/acetic acid molar ratio of 1.5 is often considered as an indicator of good operation of the microbial fermentation process [16]. Since hydrogen and its by-products, acetic and butyric acid, are easier to degrade, promoting their corresponding pathways could result in an improvement of methanogenesis kinetics [19]. Consequently, the accumulation of non-hydrogen metabolites could lead to methanogenesis inhibition, and conditions favoring AD inhibition should be avoided [20]. The feasibility of treating wheat straw, which is a model of lignocellulosic residues, in dry AD technology was previously demonstrated [9,21]. The objective of this study is to investigate the impact of TS content on microbial fermentation pathways of wheat straw, from wet to dry conditions.

**Table 1:** Main balance reactions presented in anaerobic microbial fermentation

**Metabolic pathway**	**Equation number**	**Type of fermentation**	**Reaction**	**Informative products**
Hydrogen-producing	1	Glucose fermentation	*C*_6_*H*_12_*O*_6_ + 2*H*_2_*O* → 2*CH*_3_*COOH* + 2*CO*_2_ + 4*H*_2_	Acetic acid, hydrogen
Hydrogen-producing	2	Glucose fermentation	*C*_6_*H*_12_*O*_6_ → *CH*_3_*CH*_2_*CH*_2_*COOH* + 2*CO*_2_ + 2*H*_2_	Butyric acid, hydrogen
Hydrogen-producing	3	Propionic acid fermentation	*CH*_3_*CH*_2_*COOH* + *H*_2_O → *CH*_3_*COOH* + *CO*_2_ + *H*_2_*O* + *H*_2_	Acetic acid, hydrogen
Hydrogen-consuming	4	Homoacetogenesis	2*CO*_2_ + 4*H*_2_ → *CH*_3_*COOH* + 2*H*_2_*O*	Acetic acid
Hydrogen-consuming	5	Propionic acid production	*C*_6_*H*_12_*O*_6_ + *H*_2_ → 2*CH*_3_*CH*_2_*COOH* + 2*H*_2_*O*	Propionic acid
Other metabolism	6	Propionic acid and ethanol consumption	*CH*_3_*CH*_2_*OH* + *CH*_3_*CH*_2_*COOH* → *CH*_3_*CH*_2_*CH*_2_*CH*_2_*COOH* + *H*_2_*O*	Valeric acid
Other metabolism	7	Butyric acid and ethanol consumption	*CH*_3_*CH*_2_*OH* + *CH*_3_*CH*_2_*CH*_2_*COOH* → *CH*_3_*CH*_2_*CH*_2_*CH*_2_*CH*_2_*COOH* + *H*_2_*O*	Caproic acid
Other metabolism	8	Glucose fermentation	*C*_6_*H*_12_*O*_6_ → 2*CH*_3_*CH*_2_*OH* + 2*CO*_2_	Ethanol
Other metabolism	9	Glucose fermentation	*C*_6_*H*_12_*O*_6_ → 2*CH*_3_*CHOHCOOH* + 2*CO*_2_	Lactic acid

Hydrogen-producing, hydrogen-consuming, and other metabolites are produced during acidogenesis and reveal the use of the substrate. For a more efficient methanogenesis, hydrogen-producing pathways should be favored. Adapted from Guo *et al*. [16], Valdez-Vazquez and Poggi-Varaldo [17], Cord-Ruwisch *et al*. [22], and Stadtman *et al*. [23].

## Results and discussion

### Effect of total solids (TS) content on acidogenesis microbial activity

#### Lignocellulose characterization

Wheat straw had an initial TS content of 95.6 ± 0.7% and a volatile solids (VS) content of 88.9 ± 0.7%. Wheat straw fiber analysis indicated the following fractionation (based on TS content): 9.4 ± 0.6% of soluble fraction, 33.0 ± 1.1% of hemicellulose, 43.7 ± 1.3% of cellulose, and 6.4 ± 0.8% of lignin. According to drying test results, the critical water content of the straw w_c_ was estimated at 2.4 ± 0.2 g_._g_TS_^-1^, which corresponds to a critical TS content of 29.4 ± 1.6% g_._g^-1^[24]. Production of gaseous compounds was monitored throughout the fermentation process in the reactors operated at six different TS contents. In all conditions, production of hydrogen and carbon dioxide ended after 11 days of operation.

#### Production of gaseous compounds

**Figure 1:**
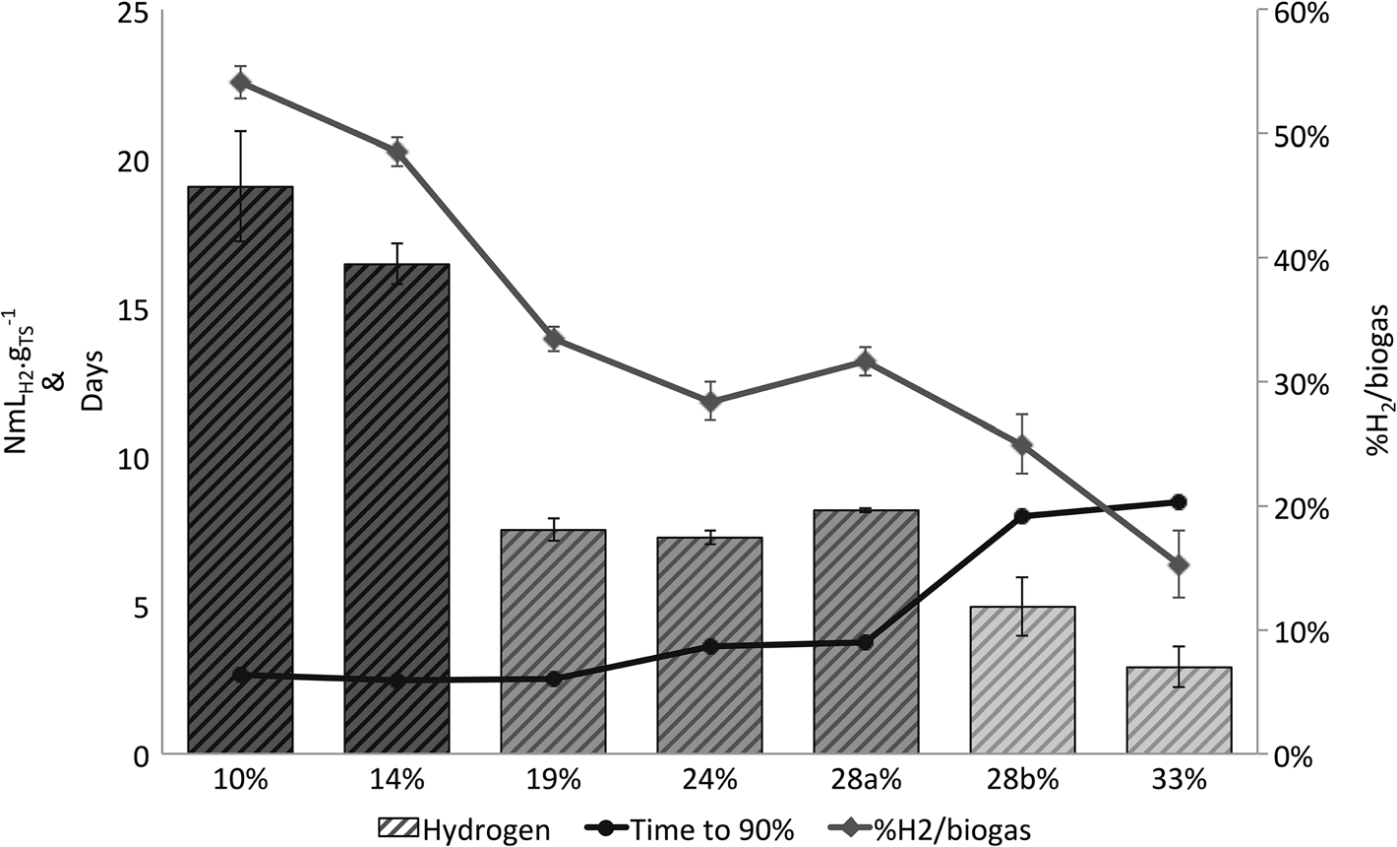
**Biohydrogen production for the six TS contents tested.** Results are expressed in terms of maximal volume, percentage of hydrogen produced, and time to achieve 90% of the total hydrogen production. These fermentation performances distinguished three groups of TS content. TS, total solids.

Production of gaseous compounds globally decreased with the increase of TS content from more than 35 NmL.g_TS_^-1^ for 10 and 14% TS, between 22 and 26 NmL.g_TS_^-1^ from 19 to 28% TS, and less than 20 NmL.g_TS_^-1^ for 28 and 30% TS. Based on kinetics and production of gaseous compounds, two distinct behaviors within the four replicates were observed at 28% TS: two replicates (named 28a% TS) had a similar trend to the experiments carried out at lower TS values, while two other replicates (named 28b% TS) behaved as at higher TS content. Other indicators are presented in Figure 1 to differentiate the conditions: the time to achieve 90% of the total hydrogen and carbon dioxide production, the hydrogen content (ratio H_2_/gaseous products), and the biohydrogen yield. The time to achieve 90% of the hydrogen production was affected by the TS content: it was less than 4 days between 10% and 28a% TS (fast reaction rate), and more than 8 days between 28b% and 33% TS (slow reaction rate). Moreover, the hydrogen content was strongly impacted and significantly decreased with the increase of TS content, from about 55% of hydrogen at 10% TS to 15% at 33% TS. Hydrogen production was lowered by the increase of TS content and three groups were distinguished (*P* value of 2.8e-13, <0.001): at 10 and 14% TS, hydrogen production was higher than 15 NmL.g_TS_^-1^, between 8 and 9 NmL.g_TS_^-1^ at 19 to 28% TS, and lower than 5 NmL.g_TS_^-1^ at 28 and 33% TS. Based on the correlation of Monlau *et al.*[25], the soluble sugar analysis indicated a hydrogen potential of 21.6 NmL.g_TS_^-1^. The hydrogen potential of the wheat straw estimate was higher than the 17.8 NmL.g_TS_^-1^ of hydrogen obtained at 10% TS. Even though the hydrogen potential is higher than generally reported in the literature (between 5 and 10 NmL.g_TS_^-1^[26,27]), the results suggest that only the soluble phase of the substrate was degraded.

#### Conversion into metabolites

**Figure 2:**
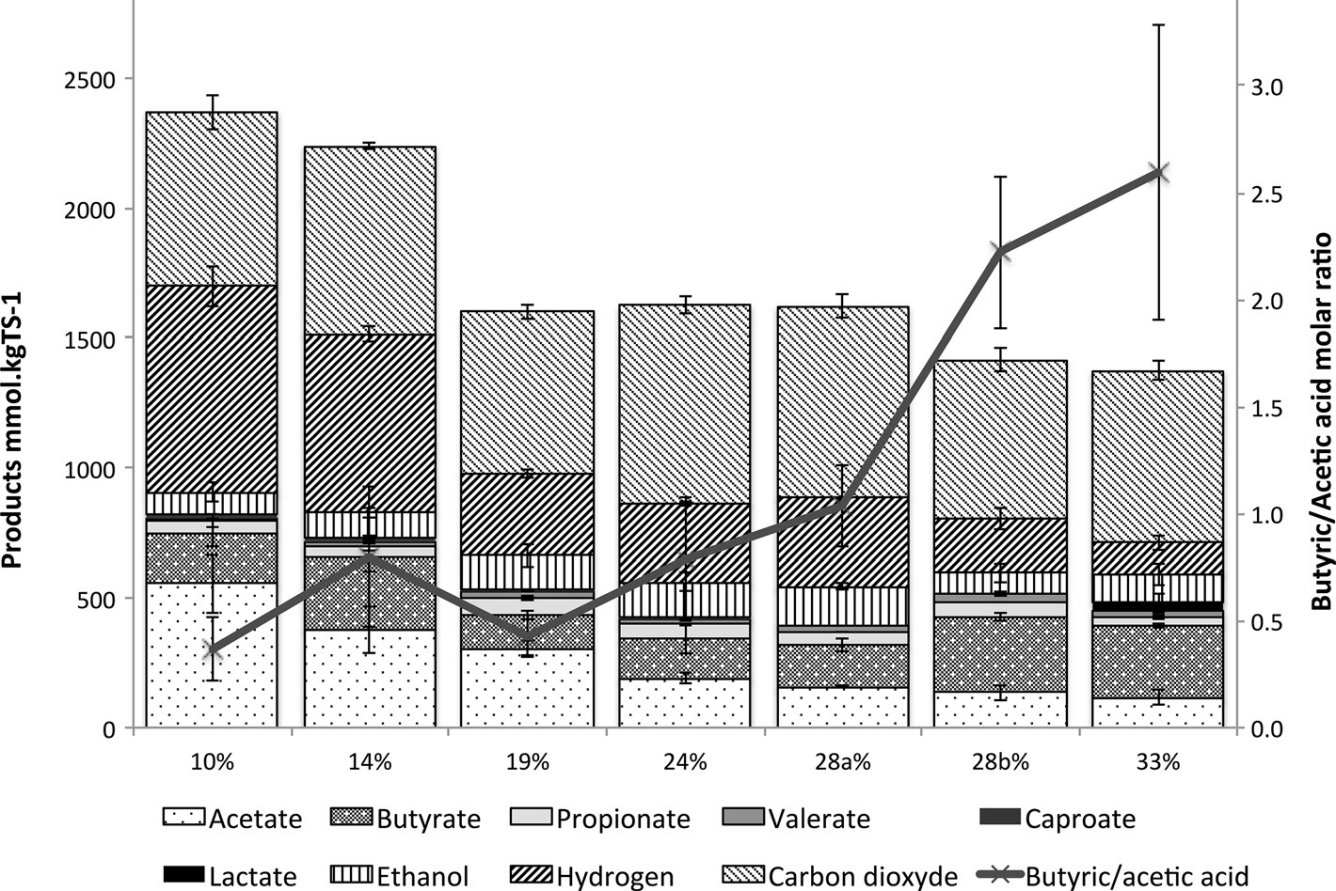
**Distribution of fermentative products and butyric/acetic acid ratio for the six TS contents tested.** These metabolites were measured after 11 days of reaction, expressed in mole unit, and reported on the initial TS content of the straw. The sum of fermentative products indicates the straw conversion yield. The butyric/acetic acid ratio indicated a metabolic shift that occurred at 28% TS. TS, total solids.

In addition to production of gaseous products, soluble fermentative end-products were simultaneously produced. Total concentration of soluble metabolites increased with the increase in TS content, with 3.6 ± 0.2, 5.4 ± 0.3, 5.6 ± 0.5, 6.4 ± 1.0, 8.0 ± 0.4, 9.8 ± 1.2, and 11.9 ± 1.4 g.L^-1^ of soluble phase at 10, 14, 19, 24, 28a, 28b, and 33% TS, respectively. The decrease of water content leads to high apparent metabolite concentrations. These concentrations are theoretically highly inhibitory for methanogenic activity, even though they are classically observed with no inhibition in dry AD [1]. The overall final pH remained constant around 5.5 ± 0.1 for all conditions despite the high concentration of soluble products. This pH was within the range of optimal pH for dark fermentation (between 5 and 6) [16,28], because of the presence of the MES buffer. Therefore, in this experiment, the absence of inhibition due to the potential acidification of the medium was likely due to the presence of a buffer. In Figure 2, the molar concentration of end-products (H_2_, CO_2_, VFA, ethanol, and lactate) was normalized by the amount of TS in the medium. The corresponding data are presented in Additional file 1. The sum of all these products, after normalization, indicates the reaction advancement of the microbial conversion of wheat straw. According to the TS content, three groups were distinguished based on substrate conversion advancement (*P* value of 5.7e-12, <0.001): 10 and 14% TS with approximately 2,288 ± 168 mmol.kg_TS_^-1^ of fermentative products, 19 to 28a% TS with approximately 1,610 ± 41 mmol.kg_TS_^-1^, and finally 28b and 33% TS with approximately 1,389 ± 38 mmol.kg_TS_^-1^.

Table 2 shows the distribution of metabolites regarding the three following categories: gaseous products, soluble products associated with hydrogen production (acetic and butyric acid), and soluble products leading to no hydrogen production (other VFAs, ethanol, and lactate). For all the conditions, the proportion of gaseous products (H_2_ and CO_2_) was in the same order of magnitude, between 57 and 67% of the sum of metabolites. However, as revealed by the hydrogen/carbon dioxide ratio, three groups were distinguished (*P* value of 2.1e-11, <0.001): 10 and 14% TS with a hydrogen/carbon dioxide molar ratio of 1.06 ± 0.14, 19 to 28a% TS with 0.46 ± 0.05, and 28b to 33% TS with 0.23 ± 0.09. On the overall range of TS content, the hydrogen associated VFAs, that is acetic and butyric acids, represented between 20 and 32% of the sum of metabolites. However, their distribution revealed only two groups based on the butyric/acetic acid molar ratios (*P* value of 2.6e-8, <0.001): from 10 to 28a% TS with an average value of 0.69 ± 0.32, and 28b and 33% TS with a significantly higher ratio of 2.47 ± 0.59. Homoacetogenic activity was quantified by estimating a theoretical molar balance between the hydrogen produced or consumed for the production of acetic, propionic, and butyric acids from the glucose equivalent and the experimental amount of hydrogen (equation 1, 2, and 5). The difference indicated the hydrogen consumed, mainly by homoacetogenesis: 462, 312, 357, 174, 81, 303, and 359 mmol.kg_TS_^-1^ of missing hydrogen at 10, 14, 19, 24, 28a, 28b, and 33% TS, respectively. If for every condition the molar balance was in agreement with the accumulation of acetic acid, at 28b and 33% TS a lack of hydrogen was observed that could not be explained by any metabolite production. The other fermentative products (propionic, valeric, and caproic acids, ethanol, and lactate) are associated with no hydrogen-producing pathways and corresponded to less than 7% of the total fermentative products for 10 and 14% TS, and more than 13% from 19 to 33% TS. The product distribution within this category was similar in most of the operating conditions for ethanol (*P* value of 0.17, not significant) and propionic acids (*P* value of 0.45, not significant) corresponding to 25 ± 7% and 56 ± 8% of the other metabolites, respectively. The main differences of the ‘no hydrogen’ metabolism were the measurement of 17% of lactate at 33% TS (*P* value of 2e-3, <0.05) and approximately 15% of caproic acid at 10 and 14% TS (*P* value of 2e-7, <0.001), and a decrease of valeric acid quantity at 10 and 14% TS (*P* value of 7e-4, <0.05).

**Table 2:** Distribution of fermentative products for the six conditions

**Categories**	**Unit (molar %)**	**Wet (W)**	**Dry (D)**	**Highly dry (HD)**	**Similarity**	**Similarity**
		**10% ≤ TS ≤14%**	**19% < TS <28%**	**28% < TS ≤33%**	**W-D group**	**W-D group**
**Gas products**	Total products	63% ± 2	63% ± 5	57% ± 5	Similar (*P* = 0.34, ns)	Different (*P* = 1e-2*)
Hydrogen	Gas products	51% ± 3	31% ± 3	18% ± 6	Different (*P* = 3e-7***)	Different (*P* = 2e-4***)
Carbon dioxide		49% ± 3	69% ± 3	82% ± 6	Different (*P* = 3e-7***)	Different (*P* = 2e-4***)
**Hydrogen associated with VFAs**	Total products	30% ± 3	23% ± 5	29% ± 2	Different (*P* = 3e-2*)	Different (*P* = 2e-4***)
Acetic acid	Hydrogen associated with VFAs	62% ± 12	62% ± 10	29% ± 5	Similar (*P* = 0.12, ns)	Different (*P* = 5e-7***)
Butyric acid		38% ± 10	38% ± 12	71% ± 5	Similar (*P* = 0.12, ns)	Different (*P* = 5e-7***)
**Other metabolites**	Total products	7% ± 2	14% ± 2	13% ± 4	Different (*P* = 3e-5***)	Similar (*P* = 0.26, ns)
Propionic acid	Concurrent metabolite	23% ± 4	26% ± 3	26% ± 13	Similar (*P* = 0.54, ns)	Similar (*P* = 0.39, ns)
Valeric acid		7% ± 3	12% ± 3	14% ± 3	Different (*P* = 7e-4*)	Different (*P* = 2e-2*)
Caproic acid		15% ± 7	1% ± 2	0% ± 0	Different (*P* = 2e-7***)	Similar (*P* = 0.07, ns)
Lactic acid		0% ± 0	0% ± 0	12% ± 13	Similar (*P* = 0.27, ns)	Different (*P* = 2e-3*)
Ethanol		55% ± 7	60% ± 7	52% ± 10	Similar (*P* = 0.71, ns)	Similar (*P* = 0.12, ns)

The three groups of TS contents are classified in three categories: gaseous products, VFAs associated with the production of hydrogen, and other metabolites. The similarity between group wet and dry and group dry and highly dry is indicated based on ANOVA. *P* value levels of significance: * < 0.05; ** < 0.01; *** < 0.001; and ns, >0.05. D, dry; HD, highly dry; ns, not significant; TS, total solids; VFA, volatile fatty acid; W, wet.

#### Distinguishment of three acidogenesis behaviors

In this study, the increase in TS contents had two consecutive consequences on acidogenesis: a sharp reduction of 25% in wheat straw conversion between 14 and 19% TS, followed by a clear shift in metabolite distribution at 28% TS. A microbial community analysis was performed on the final media to verify the origin of the metabolite distribution. Additional file 2 shows these results in more detail. The absence of correlation between the profile and TS content could attribute the butyric acid production to a metabolic shift and not to the emergence of a new population. Based on these observations, a classification in three groups of the effect of TS content on acidogenesis is proposed: a wet fermentation group (W) for 10% ≤ TS ≤14%, a dry fermentation group (D) for 19% ≤ TS <28a%, and a highly dry fermentation group (HD) for 28b% < TS ≤33%.

#### Wet fermentation group (W)

The group W, that is 10 and 14% TS batch tests, corresponds to water-saturated conditions usually described as wet digestion for AD systems. Wet digestion is characterized by a liquid medium, which allows homogeneity in the bioreactor, full access of the degrading microorganisms to the substrate, and dilution of inhibitory products (VFAs) [15]. Under these conditions, the substrate conversion rate is mainly limited by solid hydrolysis [29]. Concerning the fermentation, group W is characterized by the highest hydrogen production (more than 15 NmL.g_TS_^-1^), a fast reaction, and a hydrogen-producing pathway-oriented metabolism (more than 50% of the gaseous products). Fermentative end-products corresponded mainly to hydrogen and carbon dioxide (approximately 63% of the total metabolites), acetic and butyric acids (approximately 30% of the total metabolites), and ethanol and propionic acids (for the last 7%). Additionally, butyric/acetic acid ratios of 0.4 and 0.8, observed respectively at 10 and 14% TS, are low when compared to the overall stoichiometric ratio of 1.5 for hydrogen production reported by Guo *et al.*[16]. These low ratios resulted from higher production of acetic acid. Since the fermentation was achieved in 4 days, which resulted in a decrease of hydrogen concentrations in headspace, this acetic acid production was probably associated with hydrogen consumption. Therefore, hydrogen-consuming pathways were likely to occur either through homoacetogenesis (low butyric/acetic acid ratio, equation 4) or the propionic acid-producing pathway (equation 5) [17]. Overall, the group W shows a microbial metabolism favorable to the synthesis of microbial by-products that are easily degradable by subsequent methanogenesis (hydrogen, acetic acid, and butyric acid) [19,20].

#### Dry fermentation group (D)

In group D, that is batch tests operated between 19 and 28a% TS, metabolite distribution and reaction rate were both similar to group W. The distribution of soluble fermentative products corresponded mainly to acetic and butyric acids (23% of total metabolites), with a butyric/acetic acid ratio ranging from 0.4 to 1.0. These ratios are consistent with literature data for dark fermentation under dry conditions [30]. Here again, such metabolite distribution resulted from the delay between metabolite analysis and fermentation achievement (4 days). The decrease of hydrogen content in gas phase and an increase of carbon dioxide production were also observed in group D. Both observations, hydrogen consumption and low butyric/acetic acid ratio, suggested a significant homoacetogenesis. The homoacetogenic activity was of a lower proportion than in group W experiments: 17% ± 4 of the total straw conversion against 8% ± 4. Therefore, the decrease of hydrogen content can be explained by higher production of concurrent metabolites (30% more), which conducts to a direct consumption of hydrogen to generate propionic acid (equation 5) or the production of carbon dioxide and ethanol with no hydrogen (equation 8). Therefore, both ethanol and propionic acid can explain the decrease of the hydrogen/gaseous product ratio. Microorganisms involved in the production of ethanol and propionic acid are known to be more resistant to environmental stress than hydrogen-producing bacteria [18,31]. The decrease of substrate conversion yield of 30% between groups W and D (1,300 down to 900 mmol.kg_TS_^-1^ of fermentative product) was likely caused by the decrease of substrate accessibility due to the reduction of water content. Indeed, switching from wet to dry conditions makes it more difficult for microorganisms to access solids [32]. This hypothesis is also consistent with the slight changes of metabolisms observed between groups W and D, and the decrease of performances generally observed in dry digestion processes [6]. The limit between the two groups occurred between 14 and 19% TS in this study and is consistent with literature data, although this limit has not been clearly defined with values varying between 15 and 20% TS [3,8,9]. In the present study, no clear threshold value could be determined and further studies are required by refining the TS content between 14 and 19% TS.

#### Highly dry fermentation group (HD)

Group HD, that is 28b and 33% TS batch tests, was characterized by very low hydrogen production, low hydrogen/gaseous product ratio, and slow fermentation rates requiring 8 days to reach the maximum hydrogen productivity. The substrate conversion was reduced by approximately 40% of group W and 15% of group D. Thus, by reducing the water content, its effect was increased in group HD, with a slight increase in ethanol and propionic acid production. This observation could partly explain the reduction of hydrogen content in gas phase (only 18%) by a higher production of carbon dioxide. If the amount of acetic acid is similar to group D, a very high concentration of butyric acid was measured [26]. Since every mole of butyric acid produced is associated with the production of two moles of hydrogen (equation 2), the quantity of hydrogen measured is lower than expected (288 mmol.kg_TS_^-1^ of butyric acid against 163 mmol.kg_TS_^-1^ of hydrogen). Although the existence of hydrogen-consuming pathways that could have redirected microbial activity (equation 4 and 5), the low quantity of acetic and propionic acids fails to explain the occurrence of high concentration of butyric acid concomitant to a low hydrogen yield. These observations may suggest a butyric pathway with no hydrogen production [8,10]. Such a pathway is rarely described in the literature and results from a complex chain of reactions. For example, a rare pathway constituting of acetic acid and glucose consumption to produce butyric acid was observed in acidified thermophilic condition [33]. The important decrease of water according to the increase of TS content could explain such a metabolic shift occurring under these very particular conditions by an effect on the medium acidity.

In addition, the value of 28% TS corresponded to a critical TS content. Two duplicates (28a%) behaved like experiments in group D (19 and 24% TS), while the two others (28b%) were classified in group HD. Interestingly, similar shifts were previously reported and discussed in studies dealing with organic solids AD [8,10]. Even though the tested substrate differs (cardboard versus urban waste), these experimental works identified a threshold value of approximately 30% TS, beyond that AD was inhibited by high VFA accumulation. Abbassi-Guendouz *et al.*[12] found the emergence of specific bacterial communities for highly dry AD (*Clostridium*) and specific *Archaea* for groups W, D, and HD. To explain this phenomenon, Staley *et al.*[10] suggested that the role of local spatial heterogeneities is essential, as proposed elsewhere by Martin [32]. These authors assumed that the reduction in water content could create high local concentrations of inhibitory products leading to depleted zones with no microbial activity. However, such niches were not observed at a centimeter scale [10]. It was proposed that these depleted areas could exist but at microscopic scale [32]. Based on experimental and modeling approaches, a similar threshold of 30% TS was observed with two distinct behaviors where semi-liquid and dry conditions (10 to 30% TS) were limited by microbial hydrolysis rates, and highly dry digestion (30 and 35% TS) was limited by liquid/gas mass transfers [8]. According to drying test results, the critical TS content was estimated at 29.4 ± 1.6% (ratio in g_TS._g^-1^). Therefore, at higher TS contents, no free water was available for wheat straw degradation. Interestingly, the limit between the dry and highly dry group [8] is consistent with the critical TS content shown in this study with two different substrates and batch reactors operated under different conditions, for example no pH buffering. This value may be the limit above which free water appears to be limiting in the medium. The highly dry fermentation might be affected by a lack of free water available for microbial activity with consequences in microbial metabolisms, as shown in this study. In solid-state fermentation, Orzua *et al.*[24] suggested that the free water content is an important parameter impacting both biological and physical mechanisms of the system [15,32]. Since the lack of free water appeared only for TS content higher than 29%, extracellular enzymatic activity might remain active [34]. Further studies are required to verify whether this critical TS content may correspond to the maximum TS content to be applied for efficient acidogenesis on organic waste.

## Conclusions

**Figure 3:**
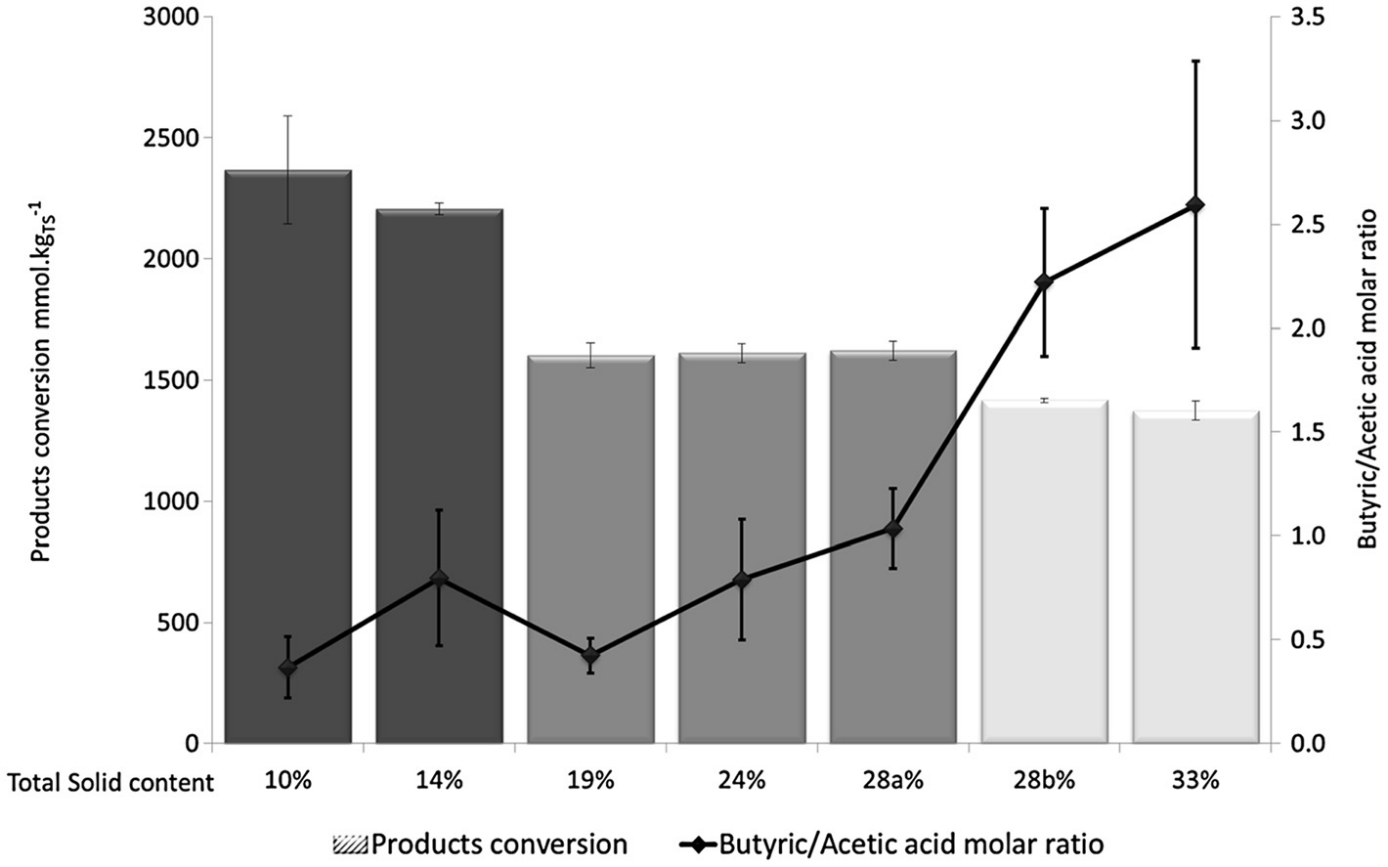
**Summary illustration of the observations of the experiment.** The product conversion corresponds to the sum of metabolites and gaseous products, which indicated the substrate conversion. If a reduction of substrate conversion occurred when TS content increased, a metabolic shift was only identified at 28% TS. TS, total solids.

The present study investigates the effect of TS content on wheat straw acidogenesis from wet to highly dry conditions (10 to 33% TS) (Figure 3). Compared to wet conditions, dry media faced heterogeneities responsible for a decrease of substrate conversion yield without modification of the metabolic pathways. However, a metabolism shift unfavorable for hydrogen production was observed at 28% TS. It was supposed that this clear limit is linked to the decrease of free water in the media. This work concludes that fermentation of lignocellulosic waste in dry conditions could be optimized in order to produce hydrogen or valuable VFAs.

## Methods

### Substrate

Wheat straw (*Triticum aestivum*) was harvested in July 2010 at an organic farm located in the Hérault region of France. A bale was homogenized manually, aliquoted in plastic bags, and stored at ambient temperature. The straw was then milled at 1 mm and sieved between 0.4 and 1 mm. The Van Soest fractionation was performed with the fiberbag system (Gerhardt, Crailsheim, Germany). The proportion of free and bound water was assessed with a drying test of wheat straw, according to the protocol proposed by García-Bernet *et al.*[35]. This method identifies the moisture content when the water evaporation kinetic starts to decrease. This point, marking the transition between bound to free water, is defined as the critical water fraction w_c_ (water content expressed in g.g_TS_^-1^).

### Inoculum source

Four different digestates were sampled in AD plants treating organic waste to obtain a high microbial diversity in the initial anaerobic inoculum. A mix of inocula was prepared to equalize the contribution of each inoculum source (25% in VS basis). Two digestates were collected from two upflow anaerobic sludge blanket (UASB) digesters treating effluents from a sugar factory or sludge from a wastewater treatment plant under mesophilic and thermophilic conditions, respectively. Two other digestates were sampled in two dry anaerobic digesters treating the organic fraction of municipal waste and operated under mesophilic and thermophilic conditions. The digestates were centrifuged at 3,000 *g* during 15 minutes at 4°C and the liquid phase was collected as inoculum. A thermal treatment (90°C, 15 minutes) was applied to the inoculum to eliminate any methanogenic activity [17,26]. It had a pH of 8.95 and composed of 2.4% TS and 1.5% VS.

### Acidogenesis batch tests

Batch tests were operated in four replicates, with six different TS contents ranging from wet to dry conditions: 10, 14, 19, 24, 28, and 33 g_TS_.g^-1^. In each 600 mL flask, the medium was composed of: 21.4 ± 0.1 g of straw, 69.3 ± 1.4 mL of inoculum, 16.0 ± 0.3 g of 2-(N-morpholino) ethanesulfonic acid (MES) buffer, and 10 mL of a 3.2% NaOH solution to maintain a pH at 5.7 ± 0.1 and 4.1 ± 0.1 mL of a trace element solution with the following composition: 1.5 g.L^-1^ of FeCl_2_,H_2_O; 62 mg.L^-1^ of H_3_BO_3_,H_2_O; 117 mg.L^-1^ of MnSO_4_,H_2_O; 26 mg.L^-1^ of CoCl_2_,6H_2_O; 120 mg.L^-1^ of ZnCl_2_; 28 mg.L^-1^ of NiCl_2_,6H_2_O; 38 mg.L^-1^ of CuCl_2_,2H_2_O; 31.8 mg.L^-1^ of NaMoO_4_,2H_2_O; and 4.4 mL of HCL 37%. Distillated water addition was calculated by mass balance on both TS and VS contents (including straw, inoculum, buffer, and solution addition) in order to obtain the required TS contents: 259.2 ± 1.9, 130.3 ± 1.0, 67.6 ± 0.7, 30.6 ± 0.3, 5.1 ± 0.1, and 0.0 ± 0.0 mL for 9.5, 14.2, 18.8, 24.0, 28.1, and 33.2% TS, respectively. The batch tests were then incubated at 35°C ± 1 for 11 days.

### Gaseous products quantification and analysis

All batch reactors were connected to a multiplexed R3000 micro-gas chromatograph (μGC) with two analytical capillary columns (SRA instrument, Marcy l’Etoile, France) to monitor gas production on line. The first column was dedicated to carbon dioxide analysis and corresponded to a 5Ǻ molecular sieve (10 m length and 0.32 mm diameter) with argon as carrier gas at a pressure of 30 PSI. The second column dedicated to oxygen, hydrogen, nitrogen, and methane analysis was a PLOT Q (8 m length and 0.32 mm diameter) with helium as carrier gas (20 PSI). The injector and column temperatures were 90°C and 80°C, respectively. The detector was a micro-thermal conductivity detector (μTCD). Multiplexing the channels allowed the simultaneous connection of 28 batch tests with a measure of the total gas production every 4 hours, by pressure measurement. To maintain a constant pressure in headspace, the gas composition was evaluated by sampling only when pressure was higher than 1.1 bars.

### Analytical procedure

Reactors were sampled at the beginning (after the inoculation phase) and the end of each experiment. Samples were homogenized with a magnetic mixer (10 and 14% TS series) or by hand-shaking (19 to 33% TS series). In order to extract the liquid fractions of the reactors a gravimetric dilution to 5% TS was performed. The liquid phase was then extracted after filtration with a microfiber filter GF/D (2.7 μm Whatman, Kent, UK). The pH was measured immediately after dilution. VFAs were measured with a gas chromatograph Varian 580 (Palo Alto, CA, USA) composed of an Elite-FFAP Crossbond Carbowax 15 m column connected to a flame ionization detector (FID) at 280°C. Nitrogen at 6 mL.min^-1^ was used as carrier gas. Main fermentation metabolites were analyzed after filtration at 0.2 μm by HPLC equipped with an HPX-87H column (Bio-Rad, Hercules, CA, USA) at 35°C. The eluting solution corresponded to a 0.005 M H_2_SO_4_ water solution at a flow rate of 0.4 mL.min^-1^.

### Statistical analysis

The R software (version 2.15.1) coupled with Rcmdr package version 1.8-4 was used for statistical significance measurement, using analysis of variance (ANOVA) with a *P* value at 0.05. The notations of significance levels of *P* values were: * < 0.05; ** < 0.01; *** < 0.001; and not significant (ns), >0.05.

## Abbreviations

AD: Anaerobic digestion; ANOVA: Analysis of variance; CE-SSCP: Capillary electrophoresis single strand conformation polymorphism; D: Dry fermentation; FID: Flame ionization detector; HD: Highly dry fermentation; HPLC: High performance liquid chromatography; MES: 2-(N-morpholino)ethanesulfonic acid; PCA: Principal component analysis; TS: Total solids; UASB: Upflow anaerobic sludge blanket; VFA: Volatile fatty acid; VS: Volatile solids; W: Wet fermentation; μGC: micro-gas chromatograph; μTCD: micro-thermal conductivity detector.

## Competing interests

The authors declare that they have no competing interests.

## Authors’ contributions

JCM designed and carried out the experiments, analyzed results, and wrote the manuscript. ET, RE, and CD assisted and validated the experimental design and reviewed the manuscript. NB, JPD, and JPS coordinated the project and reviewed the manuscript. All authors read and approved the final manuscript.

## Supplementary Material

Additional file 1 **Distribution of fermentative products for the six conditions tested.** The 28% TS condition is a threshold value and is split into two groups. This table provides the original data of Figure 2. Click here for file

Additional file 2 **Principal component analysis (PCA) of bacterial communities was characterized using CE-SSCP profiles including discriminant peaks.** Since the PCA shows an absence of correlation between profiles and TS content, butyric acid production can be attributed to a metabolic shift and not to the emergence of new population. Click here for file
