# Supplementary material for: Total solids content: a key parameter of metabolic pathways in dry anaerobic digestion
Source: Biotechnol Biofuels. 2013 Nov 22;6:164. doi: 10.1186/1754-6834-6-164 (PMC4176753; doi:10.1186/1754-6834-6-164)
Supplement: Additional file 1 — Distribution of fermentative products for the six conditions tested. The 28% TS condition is a threshold value and is split into two groups. This table provides the original data of Figure 2. [file 1754-6834-6-164-S1.pdf]

Metabolite production ($mmol.kg_{TS}^{-1}$)	10 %TS	14 %TS	19 %TS	24 %TS	28a %TS	28b %TS	33 %TS
Hydrogen	794 ± 77	685 ± 29	313 ± 15	302 ± 9	341 ± 3	205 ± 41	121 ± 29
Carbon dioxyde	672 ± 67	726 ± 12	624 ± 29	763 ± 34	738 ± 47	615 ± 43	662 ± 35
Acetic acid	554 ± 113	378 ± 90	305 ± 32	191 ± 22	156 ± 3	135 ± 29	114 ± 29
Propionic acid	47 ± 23	38 ± 15	66 ± 29	57 ± 5	52 ± 0	56 ± 2	29 ± 6
Butyric acid	194 ± 52	280 ± 56	127 ± 15	152 ± 60	161 ± 27	294 ± 17	281 ± 9
Valeric acid	8 ± 4	16 ± 3	30 ± 2	23 ± 2	23 ± 3	29 ± 9	23 ± 5
Caproic acid	19 ± 3	19 ± 9	3 ± 4	1 ± 3	0 ± 0	0 ± 0	0 ± 0
Ethanol	83 ± 38	99 ± 21	133 ± 45	138 ± 34	151 ± 12	79 ± 36	108 ± 42
Lactate	0 ± 0	0 ± 0	0 ± 0	0 ± 0	0 ± 0	3 ± 4	36 ± 33
