# Supplementary material for: Total solids content: a key parameter of metabolic pathways in dry anaerobic digestion
Source: Biotechnol Biofuels. 2013 Nov 22;6:164. doi: 10.1186/1754-6834-6-164 (PMC4176753; doi:10.1186/1754-6834-6-164)
Supplement: Additional file 2 — Principal component analysis (PCA) of bacterial communities was characterized using CE-SSCP profiles including discriminant peaks. Since the PCA shows an absence of correlation between profiles and TS content, butyric acid production can be attributed to a metabolic shift and not to the emergence of new population. [file 1754-6834-6-164-S2.pdf]

Microbial community signature

Principal Component Analysis (PCA) of bacterial communities CE-SSCP profiles including discriminant peaks. Triangles represent the wet group, squares represent the dry group and circles represent the highly dry group. Since there is no correlation between profiles and TS content, the butyric acid production is attributed to a metabolic shift and not to the emergence of new population.

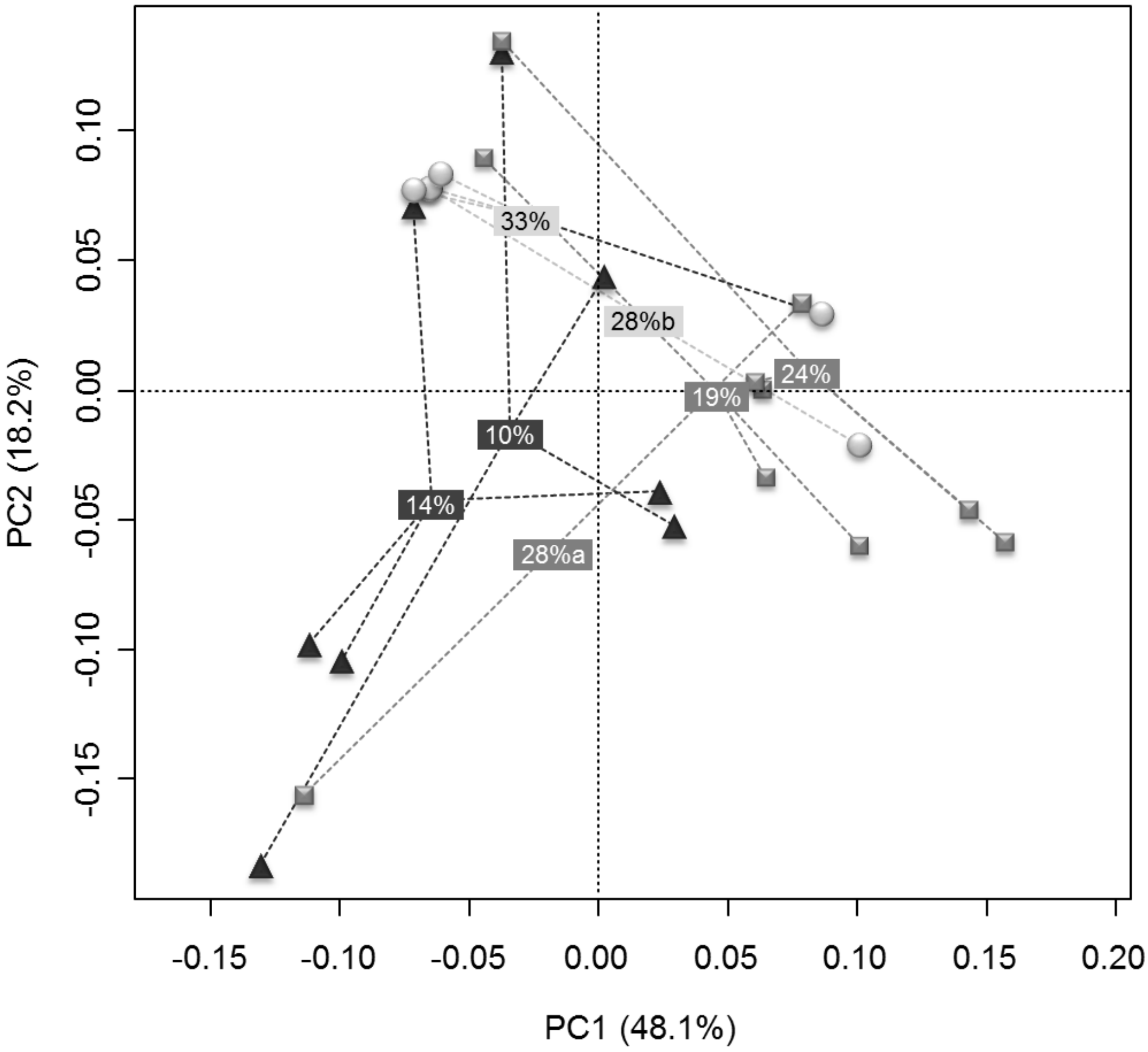

Material and method

The microbial communities of the digestate were evaluated according to their fingerprint profile. DNA extraction was performed according to the protocol present in Rousselon, *et al.*

(2004) [1]. After extraction and purification, the protocol was the same as described in Abbassi-Guendouz *et al.* (2013) [2]: PCR (Polymerase Chain Reaction) amplification of the 16S rRNA genes for bacteria followed by CE-SSCP (Capillary Electrophoresis Single-Strand conformation polymorphism). CE-SSCP profiles were analyzed, according to the protocol of Quéméneur *et al.* (2011) [3] using GeneScan software (Applied Biosystems) and the StatFingerprints R library Michelland *et al.* (2009) [4]. The CE-SSCP fingerprinting profiles were studied by Principal Component Analysis (PCA) performed with R software (version 2.15.1 [5]) using the vegan library.

References

1. Rousselon N, Delgenès J-P, Godon J-J: **A new real time PCR (TaqMan PCR) system for detection of the16S rDNA gene associated with fecal bacteria.** *Journal of microbiological methods* 2004, **59**:15–22.

2. Abbassi-Guendouz A, Trably E, Hamelin J, Dumas C, Steyer JP, Delgenès J-P, Escudié R: **Microbial community signature of high-solid content methanogenic ecosystems**. *Bioresource Technology* 2013, **133**:256–262.

3. Quéméneur M, Hamelin J, Latrille E, Steyer J-P, Trably E: **Functional versus phylogenetic fingerprint analyses for monitoring hydrogen-producing bacterial populations in dark fermentation cultures**. *International Journal of Hydrogen Energy* 2011, **36**:3870–3879.

4. Michelland RJ, Dejean S, Combes S, Fortun-Lamothe L, Cauquil L: **StatFingerprints: a friendly graphical interface program for processing and analysis of microbial fingerprint profiles.** *Molecular ecology resources* 2009, **9**:1359–63.

5. Team RDC: **R: A language and environment for statistical computing**. *R Foundation Statistical Computing* 2008, **1**.
